# Oxidative stress markers in saliva and periodontal disease status: modulation during pregnancy and postpartum

**DOI:** 10.1186/s12879-015-1003-z

**Published:** 2015-07-08

**Authors:** Pınar Gümüş, Gülnur Emingil, Veli-Özgen Öztürk, Georgios N. Belibasakis, Nagihan Bostanci

**Affiliations:** Department of Periodontology, School of Dentistry, Ege University, İzmir, Turkey; Department of Periodontology, School of Dentistry, Adnan Menderes University, Aydın, Turkey; Section of Oral Microbiology and Immunology, Institute of Oral Biology, Center of Dental Medicine, University of Zürich, Plattenstrasse 11, 8032 Zürich, Switzerland

**Keywords:** Pregnancy, Saliva, Periodontal disease, Oxidative stress, Reactive oxygen species

## Abstract

**Background:**

Periodontal diseases may affect local and systemic inflammation, and reactive oxygen species (ROS) levels. This systemic health burden could compromise the outcome of pregnancy in expectant mothers. The aim of the present study was to evaluate oxidative stress markers, including glutathione peroxidase (GPx), thiobarbituric acid-reactive substances (TBARS) and 8-hydroxy-2’-deoxyguanosine (8-OHdG), and total bacterial loads in the saliva of pregnant and postpartum women, and to investigate their association with periodontal disease severity.

**Methods:**

A total of 187 women were originally recruited for this case–control study, assigned to the following groups a) pregnant group, b) postpartum group: the pregnant group re-evaluated 6 months after giving birth, c) control group: systemically healthy and non-pregnant women. The levels of the studied oxidative stress markers in saliva were measured by commercially available kits.

**Results:**

The levels of salivary 8-OHdG were significantly elevated in the pregnant, compared with the control group. Although salivary 8-OHdG levels slightly decreased after giving birth (postpartum group), the difference did not reach significance. In contrast, the activity of antioxidant enzyme GPx in saliva was significantly lower in the pregnant than the control group. Although no differences in lipid peroxidation (represented by TBARS) were observed between the pregnant and control groups, after giving birth TBARS levels were significantly lowered. Only in the postpartum and control groups did clinical measurements of periodontal disease severity correlate with oxidative stress markers. Interestingly, there were no such correlations with TBARS in the pregnant and postpartum groups.

**Conclusions:**

The present study shows changes in the oxidant/antioxidant balance in saliva during pregnancy and after birth, which may be affected by periodontal health status in the latter case. Whether this is associated with adverse pregnancy outcomes, or not, remains to be elucidated. Early identification of ROS markers in saliva may be of clinical value in the periodontal management of pregnant women.

## Background

Periodontal diseases are a heterogeneous group of infectious/inflammatory diseases that may consequently result in tooth loss. A local elevation of pro-inflammatory mediators is caused in the periodontal tissues, which may lead to reversible inflammation (gingivitis) or to irreversible local tissue destruction (periodontitis) [5]. This local inflammatory response is initiated upon stimulation by oral bacteria in a form of polymicrobial biofilms growing on the tooth surface [39].

Significant links have been elucidated between local inflammatory periodontal disease and systemic conditions, such as diabetes mellitus and a higher risk of preterm low-birth weight babies (PLBW) [29]. In this line, many studies supported a possible bi-directional relationship between periodontal disease and pregnancy [6, 26]. During pregnancy, the occurring physiological changes affect the maternal immune system. This may reflect on the clinical presentation of both systemic and local infections, such as periodontal diseases [6, 26]. Reciprocally, it has been discussed that periodontal diseases are strongly associated with adverse pregnancy outcomes [1, 42], supported by studies showing that PLBW are more common among individuals with periodontal disease than health [22, 24]. However, others have failed to find such associations [7, 9, 17, 25].

One of the possible hypotheses to explain the potential mechanism underlying this interaction could be alteration in the oxidative stress-mediated inflammatory pathways in periodontal disease and PLBW [6, 26]. Oxidative stress represents an increase in the production of oxidants and/or a decrease in protective antioxidants. Oxygen is an essential element of aerobic life and oxidative metabolism and represents a principal source of energy, but when partially reduced, it generates reactive oxygen species (ROS) [27]. Hence, an unbalanced equilibrium between radical and non-radical ROS can damage periodontal tissues by a variety of mechanisms, including peroxidation of lipid membranes, protein inactivation, as well as stimulation of cytokine production [13, 36]. Large amounts of pro-oxidants are produced in the prolonged inflammatory responses, which occur in gingivitis and periodontitis [44]. Reversely, patients with periodontal disease often present with reduced levels of anti-oxidants [14, 34]. Therefore, periodontal disease could contribute to increased local and systemic burden of ROS in expectant mothers. Yet, multiple sources of ROS, such as stress, may account in this population. The aim of the present study was to evaluate in pregnant and postpartum women the salivary levels of oxidative stress markers in saliva, and their associations with clinical parameters of periodontal inflammation and disease severity. The measurable outcomes were the levels of glutathione peroxidase (GPx), which is an antioxidant, thiobarbituric acid-reactive substances (TBARS), a measure of ROS damage, and 8-hydroxy-2’-deoxyguanosine (8-OHdG), a measure of DNA damage, in saliva.

## Methods

### Study population

A total of 187 women participated in this study. These included 115 pregnant women (aged 19.0 to 40 years) were recruited for this study between March 2013 and March 2014, from the project of Municipality of Bornova, Dental Association and Ege University Ege University Medical School Department of Public Heatlth, Izmir, Turkey, and a control group of 72 systemically healthy non-pregnant women (aged 18.0 to 48 years) seeking dental treatment in School of Dentistry, Ege University, Izmir, Turkey. General exclusion criteria were a) any known systemic disease and, b) periodontal treatment within the last six months, c) patients having less than 10 teeth, d) smokers, and e) individuals with BMI > 30 kg/m^2^. Further, additional exclusion criteria in the pregnant group gestational diabetes mellitus and preeclampsia. Pregnant women were re-evaluated 6 months after postpartum. The study was conducted in full accordance with the ethical principles of the World Medical Association Declaration of Helsinki. The study was approved by the Ethics Committee of Ege University (protocol number 13–3.3/8) and conforms to the STROBE guidelines for observational studies [43]. The study protocol was explained to the participants, and written informed consent was obtained from each one of them prior to registering medical and dental histories, clinical periodontal examination and saliva sampling.

The study design included the following groups: 1) pregnant women (*n* = 115) who were in their second trimester (weeks 16–24) or third trimester (weeks 25–34), 2) postpartum women (*n* = 115) who were evaluated 6 months after giving birth, 3) women who were systemically healthy and non-pregnant (*n* = 72) (control group).

Clinical diagnosis of periodontal health status was further performed. Patients were diagnosed with chronic periodontitis when they had ≥4 teeth in each jaw with a probing depth (PD) of ≥5 mm and clinical attachment loss (CAL) of ≥4 mm, in at least two quadrants. Diagnosis of gingivitis was assigned when bleeding on probing (BOP) was present at >50 % of all sites and PD <3 mm at ≥90 % of the measured sites, but no more than one site had PD >4 mm and CAL ≤1 mm. The periodontally healthy women exhibited BOP <20 % of the measured sites [5, 28]. All participants had ≥20 teeth present.

### Collection and processing of saliva samples

Whole saliva samples were obtained simply by expectorating into polypropylene tubes prior to clinical periodontal measurements or any periodontal intervention. This was performed during morning sessions, following an overnight fast during which subjects were requested not to drink (except water) or chew gum. The individuals were asked to rinse their mouth with tap water, before expectorating whole saliva into sterile 50 ml tubes for 5 min. The saliva samples were then placed on ice, supplemented with EDTA-free Protease Inhibitor Cocktail (Roche Applied Science, Switzerland) prior to centrifuging at 10000 x g for 15 min at 4 °C. The resulting supernatants were immediately aliquoted and frozen (−80 °C), until the analysis.

### Clinical periodontal measurements

Subsequent to saliva sampling, clinical periodontal recordings, including dichotomous (+/−) plaque index (PI), PD, CAL, and dichomotous (+/−) presence of BOP (occurring within 15 s after periodontal probing) were performed at 6 sites on each tooth present (except third molars) using a Williams periodontal probe (Hu Friedy, Chicago, IL, USA). CAL was assessed from the cement-enamel junction to the base of the probable pocket. All clinical measurements were performed by a single calibrated examiner (PG).

### Measurement of GPx activity, 8-OHdG and TBARS in saliva

The activity of GPx in saliva supernatants was determined using the GPx assay kit (HT Glutathione Peroxidase Assay Kit, Cat# 7512-100-K, Trevigen, Gaithersburg, USA) according to the manufacturer’s instructions. Oxidized glutathione is recycled to its reduced state by glutathione reductase and nicotinamide adenine dinucleotide phosphate (NADPH). The assay principle is that oxidation of NADPH to NADP is accompanied by a decrease in absorbance immediately at 340 nm every 30 s over 12.5 min. One Unit of GPx was defined as the amount of enzyme causing oxidation of 1 nM of NADPH to NADP+ per min at 25 °C.

Lipid peroxidation was measured using thiobarbituric acid-reactive substances (TBARS) (TBARS Parameter Assay Kit, Cat# KGE013, R&D, Minneapolis, USA). For precipitating interfering proteins and other substances, trichloroacetic acid (TCA) treatment was applied to the saliva samples. After 15 min incubation at room temperature, the samples were centrifuged at 21000 x g for 4 min. The resulting supernatants were incubated with thiobarbituric acid (TBA) on a thermoshaker at 97 °C for 30 min, and the resulting color was measured spectophotometrically at 532 nm. The calibration curve of 1,1,3,3-tetraethoxypropane standard solutions was used to determine the concentrations of TBA–MDA in the samples.

Salivary 8-OHdG levels were also measured by an ELISA kit (OxiSelect^TM^ Oxidative DNA Damage ELISA Kit, Cat# STA-320, Cell Biolabs, Inc. San Diego, USA), according to the manufacturer’s instructions. A volume of 50 μl of saliva or 8-OHdG standard was used for the assay. At the end-reaction, the absorbance was read spectrophotometrically at 450 nm, and the concentrations of 8-OHdG in the samples were determined by comparison against a pre-determined standard curve.

### Bacterial quantification by quantitative real-time Polymerase Chain Reaction (qPCR)

For determining the total bacterial numbers in saliva, qPCR was employed. In brief, total bacterial DNA was extracted from 300 μl saliva by the GenElute bacterial genomic DNA kit (Sigma Aldrich, Buch, Switzerland). DNA amplification and detection was performed in a qPCR System (Step One Plus, Applied Biosystems, Life Technologies, Basel, Switzerland). Twenty ng DNA were added to each well and the reaction was run using SYBR® Green PCR Master Mix (Life Technologies, Zug, Switzerland) in a StepOnePlus™ Real-Time PCR System (Applied Biosystems) with primers were designed measure all bacteria (namely universal primers) as previously described [2].

### Statistical analysis

The distribution of the data was validated by D’Agostino-Pearson omnibus normality test and statistical analysis was performed using non-parametric methods. Comparisons between all groups were made using the Kruskal-Wallis test. Statistical analyses were conducted using the statistical software (GraphPad Prism version 6.00c for Mac OS X, GraphPad Software, La Jolla California USA), and statistical significance was considered at *p* < 0.05. Correlations between parameters were analyzed by Spearman’s correlation test.

## Results

### Clinical findings: periodontal parameters

Clinical periodontal measurements and age of the subjects among study groups are outlined in Table 1. Patients in the periodontitis non-pregnant women group were significantly older than the other groups (*P* <0.001). In periodontal health, the percentage of sites with BOP was significantly lower in the control group, than pregnant and postpartum groups, whereas in gingivitis, PD was significantly lower in pregnant and post-partum, than in non-pregnant women (*p* < 0.05). In periodontitis, CAL was significantly higher in non-pregnant, than in pregnant and postpartum women (*p* < 0.05).

**Table 1:** Clinical periodontal measurements of study groups

Groups	Pregnant Healthy	Pregnant Gingivitis	Pregnant Periodontitis	Nonpregnant Healthy	Nonpregnant Gingivitis	Nonpregnant Periodontitis	Postpartum Healthy	Postpartum Gingivitis	Postpartum Periodontitis	
	N: 22	N: 75	N: 18	N: 30	N: 30	N: 12	N: 21	N: 76	N: 18	
	Median (IQR)	Median (IQR)	Median (IQR)	Median (IQR)	Median (IQR)	Median (IQR)	Median (IQR)	Median (IQR)	Median (IQR)	*p*
Age (year)	27.9 (6.5)	25.0 (4.0)	31.0 (8.5) ^a^	25.0 (4.0)	23.5 (9.0)	40.0 (10.0) ^a^	27.5 (6.0)	27.0 (8.5)	31.5 (7.3)	*p* < 0.001
PD (mm)	1.0 (1.0)	2.0 (0.0) ^b^	5.3 (1.0)	1.0 (1.0) ^c^	3.0 (1.0) ^bd^	5.0 (1.0)	1.0 (0.0) ^c^	2.0 (0.0) ^d^	5.0 (1.0)	*p* < 0.001
CAL (mm)	1.0 (1.0)	2.0 (0.0)	6.2 (1.0) ^a^	1.0 (1.0)	3.0 (1.0)	8.1 (1.0) ^ae^	1.0 (0.0)	2.0 (0.0)	5.0 (1.0) ^e^	*p* < 0.001
BOP (%)	10.0 (10.0)^f^	60.0 (60.0)	100.0 (20)	5.0 (10.0) ^fc^	70.0 (15.0) ^d^	90.0 (20.0)	10.0 (5.0) ^c^	50.0 (20.0) ^d^	80.0 (37.5)	*p* < 0.001
PI (%)	15.0 (10.0) ^f^	70.0 (50.0)	100.0 (10) ^a^	5.0 (5.0) ^fc^	65.0 (20.0) ^d^	70.0 (20.0) ^ae^	10.0 (5.0) ^c^	50.0 (25.0) ^a^	80.0 (30.0) ^e^	*p* < 0.001

*PD* probing depth, *CAL* clinical attachment level, *BOP* bleeding on probing, *PI* plaque index, *IQR* Interquartile range

^a^significant difference between non-pregnant periodontitis and pregnant periodontitis

^b^significant difference between non-pregnant gingivitis and pregnant gingivitis

^c^significant difference between non-pregnant healthy and postpartum healthy

^d^significant difference between non-pregnant gingivitis and postpartum gingivitis

^e^significant difference between non-pregnant periodontitis and postpartum periodontitis

^f^significant difference between non-pregnant healthy and pregnant healthy

### Biochemical findings: TBARS, GPx and 8-OHdG levels

**Fig. 1:**
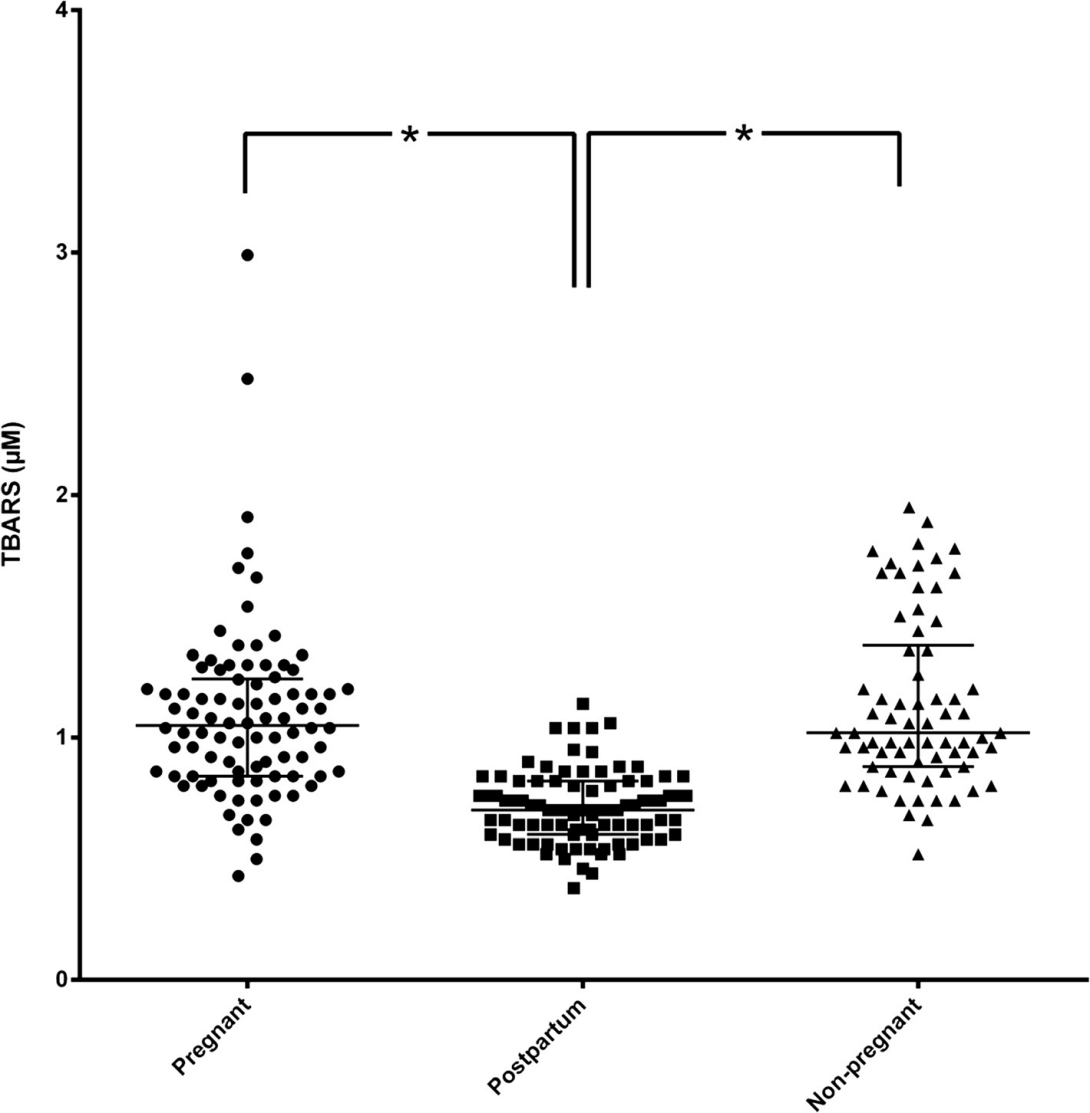
TBARS levels in the three study groups. Dot plot of TBARS levels in saliva among the three study groups (pregnant, post-partum, non-pregnant). Median and IQR were given. TBARS levels are expressed as μM

Further on, the measurement of biochemical markers for oxidative stress in saliva and serum was considered in the recruited study population, irrespective of periodontal health status. Salivary TBARS levels were significantly lower in post-partum group, than pregnant and non-pregnant groups (*p* < 0.0001), while there were no differences between non-pregnant and pregnant groups (*p* = 0.636) (Fig. 1).

**Fig. 2:**
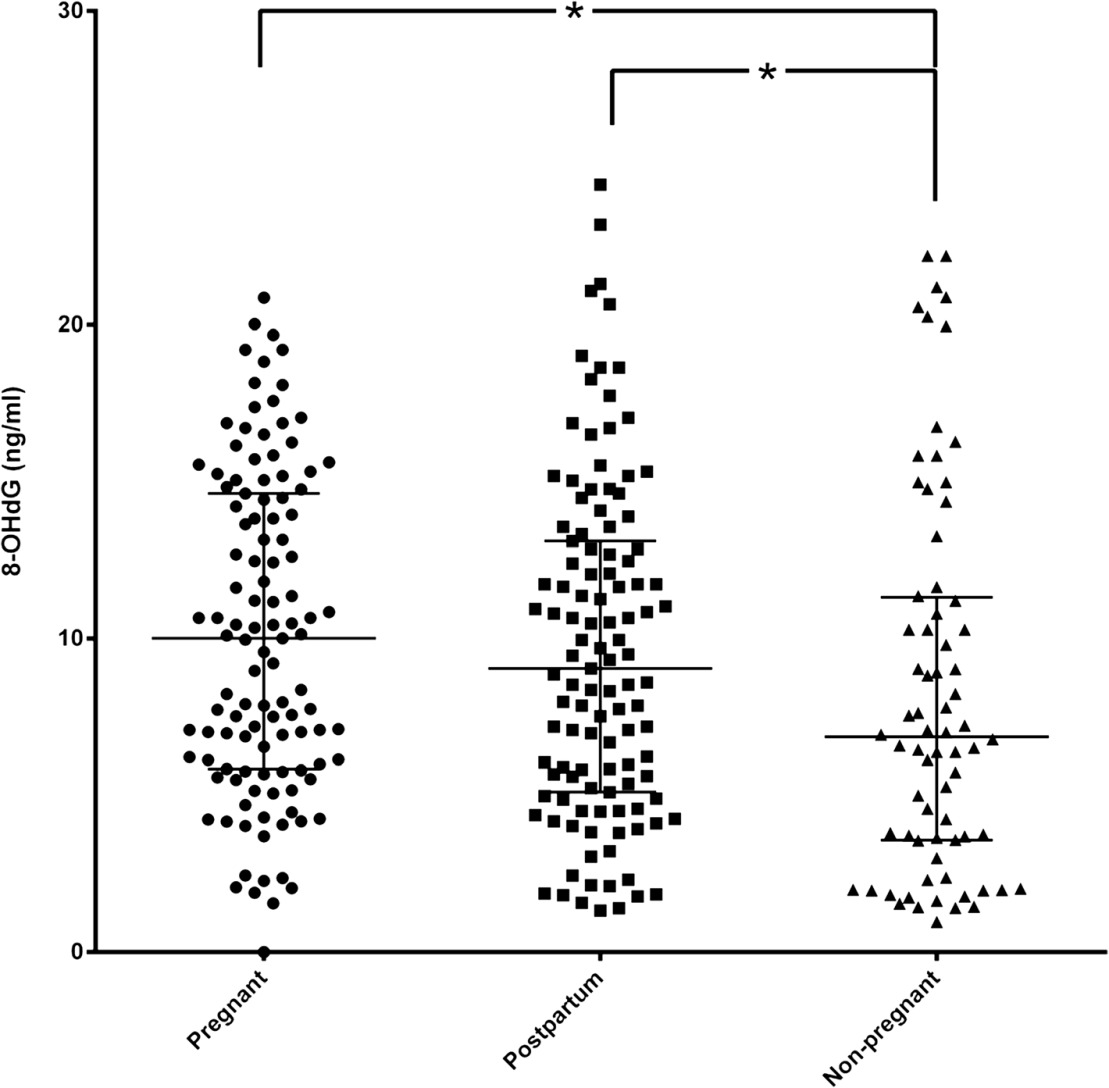
8-OHdG levels in the three study groups. Dot plot of 8-OHdG levels in saliva among the three study groups (pregnant, post-partum, non-pregnant). Median and IQR were given. 8-OHdG levels are expressed as ng/mL

Regarding 8-OHdG, the non-pregnant group exhibited significantly lower levels than pregnant and postpartum groups (*p* = 0.034, *p* = 0.0051, respectively), but there were no differences between the latter two groups (*p* = 0.3178) (Fig. 2).

**Fig. 3:**
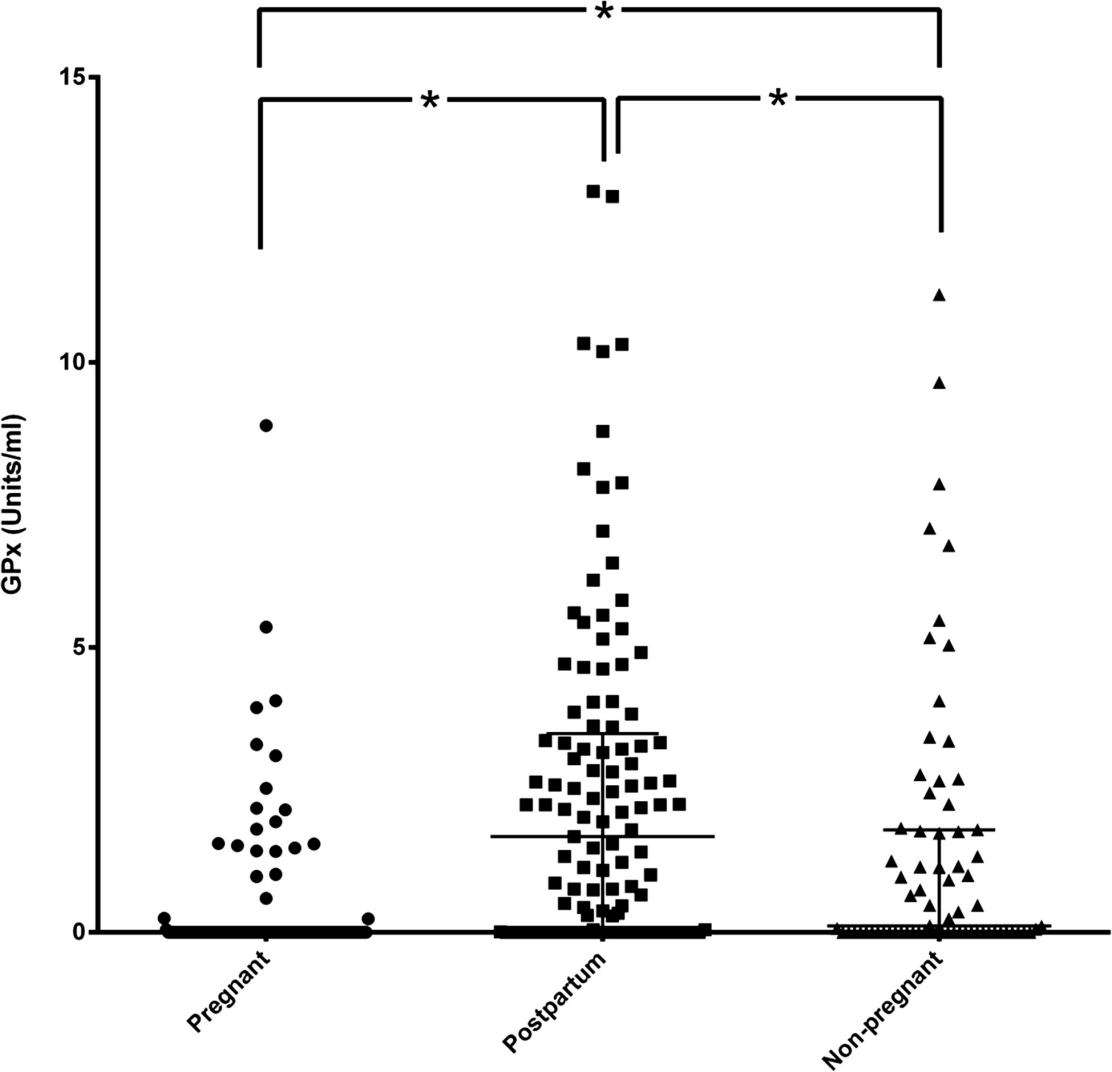
GPx levels in the three study groups: Dot plot of GPx levels in saliva among the three study groups (pregnant, postpartum, non-pregnant). Median and IQR were given. GPx levels are expressed as Units/ml

It is of interest note that the levels of GPx were below detection limits in several samples among the three study groups (pregnant; *n* = 93, post-partum; *n* = 33, non-pregnant; *n* = 33). Among detected samples, the postpartum group exhibited significantly higher GPx levels than the pregnant and non-pregnant groups (*p* < 0.0001, *p* = 0.0034, respectively). Moreover, GPx levels in the non-pregnant were significantly higher than in the pregnant group (*p* < 0.0001) (Fig. 3).

### Microbiological findings: total bacterial numbers in saliva

**Fig. 4:**
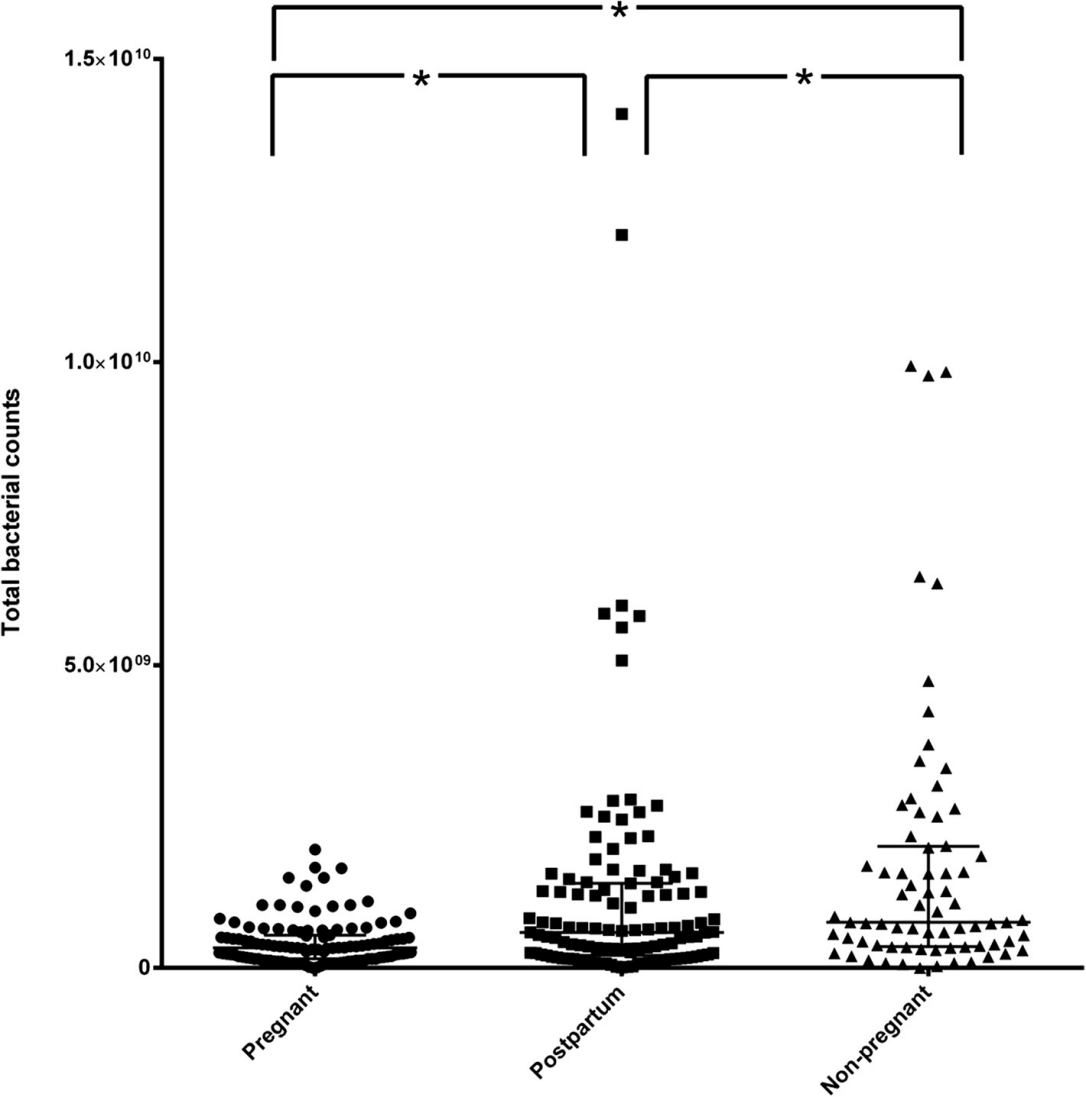
Total bacterial numbers in saliva, in the three study groups. Dot plot of total bacterial numbers per ml of saliva among the three study groups (pregnant, postpartum, non-pregnant), as defined by qPCR. Median and IQR are given

The total bacterial number per volume of saliva was also evaluated. Significant differences were detected among all pair-wise comparisons between study groups (Fig. 4). Bacterial numbers were significantly higher in the non-pregnant group, compared to the pregnant (*p* < 0.0001) or the post-partum group (*p* = 0.0158). Accordingly, the levels were also significantly higher in the post-partum compared to the pregnant group (*p* < 0.0001).

### Correlations between parameters

Finally, the associations between biochemical, microbiological and clinical periodontal parameters were evaluated in the three study groups (Table 2). Interestingly, in the pregnant women group, there were no significant correlations between biochemical, microbiological and clinical periodontal data. In the postpartum group, salivary 8-OHdG levels were positively correlated with PD and CAL, whereas salivary GPx levels were positively correlated with PD and BOP. These two oxidative stress markers were also positively correlated with total bacterial numbers. In the non-pregnant women group, positive correlations were found between GPx levels and PD, CAL, BOP or PI. On the contrary, negative correlations were revealed between TBARS and the same clinical periodontal measurements.

**Table 2:** Correlations between biochemical and periodontal data in the study groups

		Pregnant			Postpartum			Non-pregnant		
		8-OHdG	GPx	TBARS	8-OHdG	GPx	TBARS	8-OHdG	GPx	TBARS
PD (mm)	*r*	0.003	0.068	0.006	0.374	0.198	−0.105	0.070	0.475	−0.352
	p	0.974	0.468	0.955	<0001*	0.035*	0.322	0.562	<0001*	0.003*
CAL (mm)	*r*	0.003	−0.052	0.056	0.304	0.083	0.234	−0.020	0.469	−0.286
	p	0.978	0.581	0.671	<0001*	0.380	0.543	0.866	<0001*	0.016*
Plaque (%)	*r*	−0.018	−0.001	0.076	0.042	0.151	0.048	0.132	0.360	−0.435
	p	0.847	0.990	0.475	0.658	0.111	0.655	0.271	<0001*	<0001*
BOP (%)	*r*	−0.002	0.010	0.087	0.079	0.199	−0.003	0.111	0.437	−0.406
	p	0.986	0.914	0.414	0.404	0.035*	0.975	0.358	<0001*	<0001*
Total bacteria	*r*	0.073	−0.001	−0.147	0.218	0.287	−0.012	0.131	0.228	−0.175
	p	0.437	0.999	0.165	0.018*	<0001*	0.905	0.272	0.043*	0.145

* Significant correlations (*p* < 0.05)

## Discussion

A continuous balance exists in the body between ROS production and antioxidants. Overproduction of ROS and insufficiency of antioxidant mechanism results in an oxidative stress condition, which directly damages cell structures and molecules, including lipids, proteins and DNA [10]. It may also be responsible for activating key nuclear transcription factors that initiate the synthesis of pro-inflammatory mediators [15]. Conditions in which oxidative stress has been implicated include periodontitis, obesity, type II diabetes, vascular diseases and in adverse pregnancy outcomes [21]. Pregnancy is a physiological condition with affected lipid profile parameters [3] and increased oxidative stress [32]. Increased ROS production beyond the mother’s antioxidant potential leads to oxidative stress, which can affect the health of both the mother and fetus, leading to adverse pregnancy outcomes [32]. With regards to periodontal diseases, the balance between ROS and the antioxidant defense systems is postulated as one of the mechanisms responsible for periodontal tissue breakdown [15].

TBARS is a measure of ROS damage during inflammation [19], and chronic periodontitis patients are shown to exhibit higher levels of lipid peroxidation than periodontally healthy individuals [8, 30]. Periodontitis-affected gingival tissue displays significantly higher TBARS expression compared to healthy tissue, a finding that could implicate oxidative stress as a biomarker for the disease [8]. Accordingly, TBARS levels in plasma, erythrocytes, or erythrocyte membranes were found to be higher in patients with periodontitis than in healthy subjects [30]. It has also been shown that weight gain during pregnancy is associated with an increase in TBARS [4]. The present study demonstrated lower salivary TBARS levels in postpartum, compared to pregnant women. When considering the periodontal health status, no association was found between clinical measurements and TBARS salivary levels in pregnant and postpartum women. However, a negative such correlation was revealed in non-pregnant women, which might suggest periodontal health is commensurate with a reduced oxidative stress burden. In contrast, the lack of any such association in pregnant and postpartum women may denote that the physiological events of pregnancy and delivery perturb the oxidative stress status, therefore masking the putative association of TBARS with periodontal inflammation or periodontal disease severity.

Hydroxyl radical damage elicited on DNA bases can be assessed by measuring 8-OHdG levels [16]. Higher salivary and gingival crevicular fluid (GCF) levels of 8-OHdG were detected in patients with advanced periodontal destruction, and these were reported to decrease after periodontal treatment [11, 18, 35, 37, 38, 40, 41]. In the present study, non-pregnant women exhibited lower levels of 8-OHdG levels than pregnant or postpartum women. This indicates that physiological states of pregnancy and delivery increase ROS in the oral environment, as determined by 8-OHdG. This result may be well in line with earlier studies showing significantly elevated urine 8-OHdG concentrations during the third trimester of pregnancy [33]. In another study where urine 8-OHdG was measured once during 24–26 weeks of gestation, its concentrations proved to be higher in women who delivered a low birth-weight infant, although there was no association with preterm birth [23]. Interestingly, only in the postpartum group there was a positive association confirmed between this marker and periodontal disease severity (PD and CAL) and salivary bacterial load, but not with BOP. This association may indicate that following birth, oxidative stress burden may be exacerbated in the presence of periodontal disease. In this line, earlier studies have shown that salivary [35, 41] and GCF [18] 8-OHdG levels correlated with measurements of periodontal tissue destruction, particularly in chronic periodontitis [35].

A further molecule investigated in this study was GPx, an intracellular and extracellular DNA protective endogenous antioxidant [31]. GPx levels have been shown to increase by the severity of periodontal disease, while its levels decrease after nonsurgical periodontal treatment [8, 30, 44]. In pre-eclamptic women, GCF and serum GPx levels were found to be lower in the presence of periodontal disease [12]. Here, we found that GPx levels in saliva were low during pregnancy, whereas they were increased again postpartum. The increase of antioxidant levels immediately after birth may be a compensatory mechanism, particularly after a long period of exposure to increased ROS during pregnancy. When considering the periodontal disease status, no association was found with GPx in pregnant women. However, there was a positive correlation with PD, BOP and total bacterial numbers in postpartum and non-pregnant women. On the contrary, an earlier study found a negative correlation between GPx levels and PD or CAL, particularly in periodontitis [20]. The reason for this discrepancy is not clear. Yet, in the case of postpartum women, the increased GPx antioxidant levels may compensate for the sustained ROS levels during pregnancy. Hence, the association between GPx and periodontal disease severity observed here may be attributed to a disequilibrium between ROS and anti-oxidants.

## Conclusion

This is the first study to evaluate oxidative stress markers in saliva, represented by TBARS, GPx and 8-OHdG, in women during pregnancy and postpartum, and consider their association with the severity of periodontal tissue destruction. TBARS, a measure of ROS damage was elevated during pregnancy. Contrary, the antioxidant GPx was elevated postpartum, possibly to compensate for prior ROS increase. These findings clearly highlight the increased oxidative stress during pregnancy, and the dynamics developing for its compensation after birth. Yet, the association of these markers with adverse pregnancy outcomes cannot be deduced by this study. When periodontal disease status is taken into consideration, assessed by clinical periodontal parameters, there is no association with any of the studied markers during pregnancy. However, during the postpartum period positive correlations were revealed for both 8-OHdG and GPx. Hence these findings indicate that women in the postpartum period undergo a considerable oxidative stress salivary disequilibrium, and a deteriorated periodontal disease status may be a contributing factor to this.

## Abbreviations

ROS: Reactive oxygen species
GPx: Glutathione peroxidase
TBARS: Thiobarbituric acid-reactive substances
8-OHdG: 8-hydroxy-2’-deoxyguanosine
PLBW: Preterm low-birth weight babies
PD: Probing depth
CAL: Clinical attachment loss
BOP: Bleeding on probing
PI: Plaque index
qPCR: Quantitative real-time Polymerase Chain Reaction

## References

[1] Agueda A, Ramon JM, Manau C, Guerrero A, Echeverra JJ (2008). Periodontal disease as a risk factor for adverse pregnancy outcomes: a prospective cohort study. J Clin Periodontol.

[2] Akcalı A, Bostanci N, Özçaka Ö, Öztürk-Ceyhan B, Gümüş P, Buduneli N (2014). Association between polycystic ovary syndrome, oral microbiota and systemic antibody responses. PLoS One.

[3] Aksoy H, Aksoy AN, Ozkan A, Polat H (2009). Serum lipid profile, oxidative status, and paraoxonase 1 activity in hyperemesis gravidarum. J Clin Lab Anal.

[4] Ardalić D, Stefanović A, Kotur-Stevuljević J, Vujović A, Spasić S, Spasojević-Kaliomanvska V (2014). The influence of maternal smoking habits before pregnancy and antioxidative supplementation during pregnancy on oxidative stress status in a non-complicated pregnancy. Adv Clin Exp Med.

[5] Armitage GC (1999). Development of a classification system for periodontal diseases and conditions. Ann Periodontol.

[6] Armitage GC (2013). Bi-directional relationship between pregnancy and periodontal disease. Periodontol 2000.

[7] Bassani DG, Olinto MTA, Kreiger N (2007). Periodontal disease and perinatal outcomes: a case–control study. J Clin Periodontol.

[8] Borges I, Moreira EA, Filho DW, de Oliveira TB, da Silva MB, Fröde TS (2007). Proinflammatory and oxidative stress markers in patients with periodontal disease. Mediators Inflamm.

[9] Buduneli N, Baylas H, Buduneli E, Türkoğlu O, Köse T, Dahlen G (2005). Periodontal infections and pre-term low birth weight: a case–control study. J Clin Periodontol.

[10] Buonocore G, Perrone S, Tataranno ML (2010). Oxygen toxicity: chemistry and biology of reactive oxygen species. Semin Fetal Neonatal Med.

[11] Canakci CF, Cicek Y, Yildirim A, Sezer U, Canakci V (2009). Increased levels of 8-hydroxydeoxyguanosine and malondialdehyde and its relationship with antioxidant enzymes in saliva of periodontitis patients. Eur J Dent.

[12] Çanakçı V, Yıldırım A, Canakçı CF, Eltas A, Çiçek Y, Canakçı H (2007). Total antioxidant capacity and antioxidant enzymes in serum, saliva, and gingival crevicular fluid of preeclamptic women with and without periodontal disease. J Periodontol.

[13] Chapple IL (1997). Reactive oxygen species and antioxidants in inflammatory diseases. J Clin Periodontol.

[14] Chapple IL, Mason GI, Garner I, Matthews JB, Thorpe GH, Maxwell SR (1997). Enhanced chemiluminescent assay for measuring the total antioxidant capacity of serum, saliva and crevicular fluid. Ann Clin Biochem.

[15] Chapple JLC, Matthews JB (2007). The role of reactive oxygen and antioxidant species in periodontal tissue destruction. Periodontol 2000.

[16] Collins AR, Cadet J, Moller L, Poulsen HE, Vina J (2004). Are we sure we know how to measure 8-oxo-7, 8-dihydroguanine in DNA from human cells?. Arch Biochem Biophys.

[17] Davenport ES, Williams CECS, Sterne JAC, Murad S, Sivapathasundram V, Curtis MA (2002). Maternal periodontal disease and preterm low birthweight: case–control study. J Dent Res.

[18] Dede FÖ, Ozden FO, Avcı B (2013). 8-hydroxy-deoxyguanosine levels in gingival crevicular fluid and saliva in patients with chronic periodontitis after initial periodontal treatment. J Periodontol.

[19] Halliwell B, Whiteman M (2004). Measuring reactive species and oxidative damage in vivo and in cell culture: how should you do it and what do the results mean?. Br J Pharmacol.

[20] Huang P, Su T, Wang H (2000). The relationship between GPx activity in gingival fluid and clinical parameters of adult periodontitis. Hua Xi Kou Qiang Yi Xue Za Zhi.

[21] Ishikawa I (2007). Host responses in periodontal diseases: a preview. Periodontol 2000.

[22] Jeffcoat MK, Geurs NC, Reddy MS, Cliver SP, Goldenberg RL, Hauth JC (2001). Periodontal infection and preterm birth: results of a prospective study. J Am Dent Assoc.

[23] Longini M, Perrone S, Vezzosi P, Marzocchi B, Kenanidis A, Centini G (2007). Association between oxidative stress in pregnancy and preterm premature rupture of membranes. Clin Biochem.

[24] Lopez NJ, Smith PC, Gutierrez J (2007). Higher risk of preterm birth and low birth weight in women with periodontal disease. J Dent Res.

[25] Lunardelli AN, Peres MA (2005). Is there an association between periodontal disease, prematurity and low birth weight? A population-based study. J Clin Periodontol.

[26] Madianos PN, Bobetsis YA, Offenbacher S (2013). Adverse pregnancy outcomes (APOs) and periodontal disease: pathogenic mechanisms. J Periodontol.

[27] McCord JM (2000). The evolution of free radicals and oxidative stress. Am J Med.

[28] Offenbacher S, Barros SP, Beck JD (2008). Rethinking periodontal inflammation. J Periodontol.

[29] Otomo-Corgel J, Pucher JJ, Rethman MP, Reynolds MA (2012). State of the science: chronic periodontitis and systemic health. J Evid Base Dent Pract.

[30] Panjamurthy K, Manoharan S, Rajamani RC (2005). Lipid peroxidation and antioxidant status in patients with periodontitis. Cell Mol Biol Lett.

[31] Patel SP, Rao NS, Pradeep AR (2012). Effect of nonsurgical periodontal therapy on crevicular fluid and serum glutathione peroxidase levels. Dis Markers.

[32] Saker M, Mokhtari NS, Merzouk SA, Merzouk H, Belarbi B, Narce M (2008). Oxidant and antioxidant status in mothers and their newborns according to birthweight. Eur J Obst Gynecol Reprod Biol.

[33] Scholl TO, Stein TP (2001). Oxidant damage to DNA and pregnancy outcome. J Matern Fetal Med.

[34] Sculley DV, Langley-Evans SC (2003). Periodontal disease is associated with lower antioxidant capacity in whole saliva and evidence of protein oxidation. Clin Sci.

[35] Sezer U, Çiçek Y, Çanakçı CF (2012). Increased salivary levels of 8-hydroxydeoxyguanosine may be a marker for disease activity for periodontitis. Dis Markers.

[36] Sies H (1997). Oxidative stress: oxidants and antioxidants. Exp Physiol.

[37] Su H, Gornitsky M, Velly AM, Yu H, Benarroch M, Schipper HM (2009). Salivary DNA, lipid, and protein oxidation in nonsmokers with periodontal disease. Free Radic Biol Med.

[38] Sugano N, Yokoyama K, Oshikawa M, Kumagai K, Takane M, Tanaka H (2003). Detection of Streptococcus anginosus and 8-hydroxydeoxyguanosine in saliva. J Oral Sci.

[39] Sun Y, Guo QM, Liu DL, Zhang MZ, Shu R (2010). In vivo expression of Toll-like receptor2, Toll-like receptor 4, CSF2 and LY64 in Chinese chronic periodontitis patients. Oral Dis.

[40] Takane M, Sugano N, Ezawa N, Uchiyama T, Ito K (2005). A marker of oxidative stress in saliva: association with periodontally-involved teeth of a hopeless prognosis. J Oral Sci.

[41] Takane M, Sugano N, Iwasaki H, Iwano Y, Shimizu N (2002). New biomarker evidence of oxidative DNA damage in whole saliva from clinically healthy and periodontally diseased individuals. J Periodontol.

[42] Toygar HU, Seydaoğlu G, Kurklu S, Güzeldemir E, Arpak N (2007). Periodontal health and adverse pregnancy outcome in 3,576 Turkish women. J Periodontol.

[43] Von Elm E, Altman DG, Egger M, Pocock SJ, Gotzsche PC (2007). The strengthening the reporting of observational studies in epidemiology (STROBE) statement: guidelines for reporting observational studies. PLoS Med.

[44] Wei PF, Ho KY, Ho YP, Wu YM, Yang YH, Tsai CC (2004). The investigation of glutathione peroxidase, lactoferrin, myeloperoxidase and interleukin-1beta in gingival crevicular fluid: Implications for oxidative stress in human periodontal diseases. J Periodontal Res.

